# Mitochondrial matrix pH as a decisive factor in neurometabolic imaging

**DOI:** 10.1117/1.NPh.4.4.045004

**Published:** 2017-11-14

**Authors:** Patrick M. Schaefer, Diana Hilpert, Moritz Niederschweiberer, Larissa Neuhauser, Sviatlana Kalinina, Enrico Calzia, Angelika Rueck, Bjoern von Einem, Christine A. F. von Arnim

**Affiliations:** a Ulm University, Department of Neurology, Ulm, Germany; b Ulm University, Core Facility Confocal and Multiphoton Microscopy, Ulm, Germany; c University Medical School, Institute of Anesthesiological Pathophysiology and Process Engineering, Ulm, Germany

**Keywords:** NAD(P)H-FLIM, respirometry, energy metabolism, mitochondria, matrix pH, redox state

## Abstract

Alterations of cellular bioenergetics are a common feature in most neurodegenerative disorders. However, there is a selective vulnerability of different brain regions, cell types, and even mitochondrial populations to these metabolic disturbances. Thus, the aim of our study was to establish and validate an *in vivo* metabolic imaging technique to screen for mitochondrial function on the subcellular level. Based on nicotinamide adenine dinucleotide (phosphate) fluorescence lifetime imaging microscopy [NAD(P)H FLIM], we performed a quantitative correlation to high-resolution respirometry. Thereby, we revealed mitochondrial matrix pH as a decisive factor in imaging NAD(P)H redox state. By combining both parameters, we illustrate a quantitative, high-resolution assessment of mitochondrial function in metabolically modified cells as well as in an amyloid precursor protein-overexpressing model of Alzheimer’s disease. Our metabolic imaging technique provides the basis for dissecting mitochondrial deficits not only in a range of neurodegenerative diseases, shedding light onto bioenergetic failures of cells remaining in their metabolic microenvironment.

## 1 Introduction

Bioenergetic alterations and mitochondrial disturbances are prominent features in a wide range of pathologies such as cancer^1^ or neurodegenerative diseases,^2^ though these subtle changes in cellular bioenergetics between health and early disease are further masked by a high heterogeneity in mitochondrial function.^3^ It becomes most apparent in the brain, where cells cooperate closely in maintaining cellular energy metabolism.^4^ Thus, unraveling the underlying mechanisms of mitochondrial dysfunction in these pathologies requires a quantitative readout of cellular energy metabolism with high spatial resolution. A promising approach is detecting cellular redox state by imaging of nicotinamide adenine dinucleotide (phosphate) (NAD(P)H) autofluorescence.^5^ The redox couple ${\mathrm{NAD}}^{+}/\mathrm{NADH}$ is reduced in glycolysis and the tricarboxylic acid cycle and oxidized either at complex I of the mitochondrial respiratory system or by lactic acid fermentation. As only the reduced form exhibits autofluorescence, imaging of NAD(P)H intensity can be used to monitor metabolic alterations.^6^ Furthermore, it was shown that the portions of free to protein-bound NAD(P)H change along metabolic alterations with increasing free NAD(P)H in cells mainly performing glycolysis and a larger protein-bound fraction when oxidative phosphorylation is elevated.^7^ Measuring NAD(P)H autofluorescence using fluorescence lifetime imaging microscopy (FLIM) allows free and protein-bound NAD(P)H to be distinguished by their decay time.^8^ Whereas free NADH possesses a short decay time of about 400 ps, protein-bound NADH has a longer decay time of around 2500 ps.^8^^,^^9^ Consequently, NAD(P)H FLIM can be used to estimate cellular energy metabolism.

NAD(P)H intensity, spectra, and lifetime have been extensively studied in the metabolic context using inhibitors and uncouplers of the mitochondrial respiratory chain.^10^^–^^13^ Applied, it was shown that the glycolytic switch in tumor cells can be detected using NAD(P)H-FLIM^14^^,^^15^ and NAD(P)H lifetimes could be correlated to metabolic alterations during osteogenic differentiation^16^ and hypoxic microenvironment.^9^ However, NADH is also involved in several other cellular processes such as cell death or calcium signaling.^17^ In addition, NADH autofluorescence cannot be separated spectrally from NADPH autofluorescence, which is influenced by the antioxidant response to reactive oxygen species.^18^ Furthermore, the protein composition of a cell, ionic strengths, viscosity, inner filter effects, or changes in ${\mathrm{NAD}(P)}^{+}/\mathrm{NAD}(P)H$ pool sizes could influence NAD(P)H autofluorescence intensity or lifetime.^19^^,^^20^ Accordingly, Bird et al. showed a decrease in NAD(P)H lifetime in cells reaching confluence, a state when glycolysis is most likely downregulated according to the Warburg effect.^21^ Similarly, Hung et al. reported alterations in cytosolic NAD(P)H intensity depending on extracellular lactate-to-pyruvate ratio.^22^ The aforementioned influencing factors exacerbate the quantification of cellular energy metabolism using NAD(P)H autofluorescence. Thus, recently several reports describe detailed correlations of glycolysis levels and optical redox measurements.^23^^,^^24^ However, concerning mitochondrial respiration, a quantitative correlation of NAD(P)H autofluorescence and mitochondrial function is missing. Thus, the aim of our study was to correlate NAD(P)H FLIM with high-resolution respirometry to overcome any limitations due to confounding factors. This is deemed to provide a valuable, fail-safe basis to pave the way for a deeper understanding of energy metabolism on the subcellular level. Here, we could carve out the most important influencing factor for metabolic NAD(P)H FLIM, the mitochondrial matrix pH. As changes in mitochondrial membrane potential and the associated mitochondrial matrix pH often coincide with defects in the mitochondrial respiratory system, mitochondrial matrix pH is an important factor to be considered in the interpretation of NAD(P)H FLIM in matters of mitochondrial function. Setting up parallel NAD(P)H FLIM/mito-pH measurements allowed for the first time to quantitatively illustrate mitochondrial respiration.

## 2 Material and Methods

### 2.1 Cell Culture and Primary Cells

The human embryonic kidney cell line HEK293 (DSMZ no.: ACC 305/ obtained 2008) and the murine embryonic fibroblast cell line NIH-3T3 (DSMZ no.: ACC 53/ gift from Prof. Geiger) were cultured at 37°C and 5% ${\mathrm{CO}}_{2}$ in high glucose Dulbecco’s Modified Eagle’s Medium (Gibco) supplemented with 10% fetal calf serum and 1% penicillin/streptomycin. Primary astrocytes from postnatal mice (P1-P5) were prepared and cultured according to Wiesner et al.^25^

### 2.2 Electron Microscopy

HEK293 cells were seeded on pretreated sapphire disks.^26^ After 48 h, high-pressure freezing and freeze-substitution were performed.^27^ Cells were embedded in LR-Gold and cut according to Wilkat et al.^26^ A JEM-1400 transmission electron microscope (Jeol GmbH, Eching, Germany) was used at an acceleration voltage of 120 kV. Images were recorded with an image size of 2048×2048  pixel using a Veleta digital camera (Olympus Soft Imaging Solutions GmbH, Münster, Germany) and the iTEM software (Olympus Soft Imaging Solutions GmbH, Münster, Germany). A minimum of 500 mitochondria per condition were morphologically analyzed by ImageJ 1.48v (National Institutes of Health).

### 2.3 Expression Vectors

SypHer mt (Addgene #48251), GW1-Mito-pHRed (Addgene #31474), and pUltra-hot (Addgene #24130) were gifts from Nicolas Demaurex, Gerry Yellen, and Malcolm Moore, respectively. SypHer mt allows the expression of a pH-sensitive ratiometric cpYFP derivative that contains two mitochondrial matrix localization sequences at its N-terminus.^28^^,^^29^ GW1-Mito-pHRed encodes for a pH-sensitive ratiometric m-Keima derivative that contains four cytochrome C oxidase subunit VIII (Cox 8) tags to target it to the mitochondria.^30^ We cloned the pH-sensors into the lentiviral expression vector pUltra-hot to produce viruses for an efficient, moderate, and stable expression of the pH indicators in our target cells. For cloning procedure, please see SI.

### 2.4 Lentiviral Transduction System

The lentiviral expression vector pUltra-hot represents a third-generation vector that just contains the information for bacterial replication, the terminal recombination sequences, and the packaging signal. Thus, virus particles are not able to replicate without additional plasmids. To produce virus, LentiX 293T cells (Clonetech) were transfected (calcium phosphate transfection method) with psPax2, which encodes packaging proteins, pMD2.G, which codes for the envelope protein, and the respective pUltra-hot vector. Then, 6 h posttransfection, the medium was changed to avoid transfection reagent in the conditioned medium to which the virus was secreted. Forty-eight-hours posttransfection conditioned medium was collected and filtered using a 0.2-μM sterile filter (Sarstedt) and stored at −20°C.

### 2.5 High-Resolution Respirometry

Forty-eight-hour prior to respirometry, 4.5 million HEK293 cells were seeded in a $75{\text{-}\mathrm{cm}}^{2}$ flask. To synchronize the cells, the medium was changed 18 h prior to the experiment. For measurement, cells were trypsinized, counted, and resuspended in their conditioned medium to a final concentration of 1 mio cells per ml if not indicated otherwise. High-resolution respirometry was performed in an Oxygraph-2k system (Oroboros Instruments, Innsbruck, Austria) calibrated to air (gain for oxygen sensor was set to 4) with a cell culture medium. Cells were added to the two stirred (750 rpm) chambers, which were sealed to obtain a closed system. Decreasing oxygen concentration in the chambers resembled cellular oxygen consumption. First, steady-state oxygen consumption displays “routine” respiration. Subsequently, the effect of single substances on mitochondrial respiration was tested by titration of the complex I inhibitor rotenone (Rot) or the uncouplers FCCP (carbonyl cyanide-p-trifluoromethoxyphenylhydrazone) or valinomycin (Val). A stable oxygen consumption level was awaited before the next injection. Finally, the addition of 1-μM rotenone and 5-μM antimycin A blocked mitochondrial respiration completely, showing residual oxygen consumption (ROX). For testing appropriate concentrations of a complex IV inhibitor to block respiration, potassium cyanide was added after routine respiration, ending up with a concentration of 1-mM potassium cyanide. Analysis of the measurements was performed using DatLab version 5.1.0.20 (Oroboros Instruments, Innsbruck, Austria). Time intervals were drawn at the stable plateaus of oxygen flux quantifying the mean oxygen consumption of the respiratory states, which were corrected for ROX afterward. All uncouplers and inhibitors were bought from Sigma-Aldrich.

### 2.6 NAD(P)H FLIM

HEK293 cells, 3T3 cells, and astrocytes were seeded in four-chambered 35-mm dishes with glass bottom (Greiner, 627870) in a culture medium supplemented with 10% FCS, 1% P/S, and 25-mM HEPES (pH: 7.4). HEK293 cells and 3T3 cells were imaged at 37°C and atmospheric ${\mathrm{CO}}_{2}$ in their conditioned medium or in a fresh medium with the same composition despite the pH, which was altered as indicated. Primary astrocytes were imaged in Tyrodes buffer, pH: 7.4.

Lifetime imaging of NAD(P)H [nicotinamide adenine dinucleotide (phosphate)] autofluorescence was performed on a laser scanning microscope (LSM 710, Carl Zeiss, Germany) equipped with a pulsed (80 MHz, 100 fs pulse width) titanium-sapphire laser (Mai Tai AX HPDS, Spectra Physics, Germany). NAD(P)H was excited using two photons at 730 nm with a maximal power of 5 mW at the output of the objective lens and detected using a 460/60 emission filter (AHF Analysentechnik, Tübingen, Germany). Time-correlated single photon counting (TCSPC) was performed by the hybrid detector HPM-100-40 (Becker & Hickl GmbH, Berlin, Germany) coupled to the NDD port of the LSM 710. For each photon, the location within the scanning area and the time of the photon within the laser pulse period are determined using an image size of 512×512  pixel and a temporal resolution of 256 time channels within a pulse period of 12.5 ns. Collection time was set to 60 s with a pixel dwell time of ≈15  μs. An area of $132.5\times 132.5\;{\mu m}^{2}$ was scanned using an EC Plan-Neofluar 40×/1.30 oil objective.

TCSPC data were recorded using SPCM 9.6 (Becker & Hickl GmbH) and analyzed using SPCImage 5.0 (Becker & Hickl GmbH). The instrument response function was determined automatically by the software according to bH TCSPC handbook.^31^ A biexponential decay with lifetime components of 400 and 2500 ps for free and protein-bound NAD(P)H was assumed. Fixation of lifetimes was performed to improve the precision of the fitting procedure with low photon numbers, allowing a lower laser intensity and improved spatial resolution by minimizing the need for binning. The validity of this analysis was crosschecked by comparing the results with those of a free lifetime component analysis (data not shown).

The mean lifetime (τmean) of a pixel was calculated using a binning factor of 2 to 4. Fitting of the calculated lifetime curve was checked by evaluating the mean $\chi^{2}$, which was below 1.2.

Subcellular analysis of NAD(P)H lifetime was performed by calculating τmean of regions of interests (ROIs), drawn manually, in an image. At least 10 ROIs were analyzed per image. Mitochondria could be recognized as the brightest pixels as they are known to have the highest NAD(P)H concentration.^32^

After a treatment with a metabolic modifier, cells were given 5-min adaption time and then imaged within 15 min, keeping the time period similar to our reference experiments in the Oroboros Oxygraph.

### 2.7 Determination of Intracellular pH

Cells were seeded in the same way as for NAD(P)H FLIM experiments. To assess intracellular pH, cells were stained for 15 min in 1-μM BCECF-AM [2′,7′-bis-(2-carboxyethyl)-5-(and-6)-carboxyfluorescein, acetoxymethyl ester] diluted in Tyrodes buffer. Subsequently, the medium was changed to the respective cell culture medium and cells were incubated for at least 15 min at 37°C and atmospheric ${\mathrm{CO}}_{2}$ to allow the intracellular pH adjust to the conditions in the microscope. Subsequently, cells were imaged using an LSM 710 (Carl Zeiss, Germany). Per treatment, 100 cells were recorded at 525-nm emission, each being excited using 405, 458, and 488 nm. Emission ratios at the different excitation wavelengths (405/488, 458/488, and 405/458) allow for estimation of pH alterations. To assign absolute pH values to the emission ratios, a calibration according to the nigericin-high potassium method was performed.^33^ Shortly, cells were incubated in a calibration solution with 25  μM of the $K^{+}/H^{+}$ ionophore nigericin. This results in an adjustment of the intracellular pH to the pH of the calibration solution. BCECF emission ratios were measured at five different pH values ranging from 7.0 to 8.0. Within this range, the emission ratios showed a nearly linear correlation to the pH. For calculation of the intracellular pH, the three emission ratios were assigned a corresponding pH-value and the mean pH value of the three ratios was taken. Images were analyzed using Zeiss Zen2010 software.

### 2.8 Determination of Mitochondrial Matrix pH

In order to measure mitochondrial matrix pH, we used another ratiometric dye namely mito-SypHer, which was a gift from Nicolas Demaurex (Addgene plasmid # 48251). Mito-SypHer was cloned into the lentiviral expression vector pUltra-hot, which was a gift from Malcolm Moore (Addgene plasmid # 24130) (for details see Sec. 2.3). The respective virus was added to the cells resulting in an overexpression of SypHer located in the mitochondria, which was checked microscopically. Transduced cells were seeded and cultivated according to the FLIM protocol. Per experiment and per treatment, 100 cells were recorded, each being imaged with two different excitation wavelengths (405 and 488 nm). Emission was recorded at 525 nm. For calculation of the pH value corresponding to the emission ratio (405/488), nigericin-high potassium method was performed according to the BCECF calibration described above. For assessment of mitochondrial matrix pH in the amyloid precursor protein (APP)-overexpressing model and respective control cells, cells were cotransduced for MitoSypHer and APP/control vector. Subsequently, 20,000 cells were analyzed for MitoSypHer fluorescence intensity using FACS analysis (FACS Calibur). Calibration was performed using the nigericin-high potassium method mentioned above.

### 2.9 Parallel NAD(P)H FLIM/pH Imaging

For parallel imaging of NAD(P)H autofluorescence and mitochondrial and cytosolic pH, we created a lentiviral vector containing SypHer targeted to the mitochondria and pHred targeted to the cytoplasm. For cloning of the vector, please see Sec. 2.3. Addition of the virus to the cells resulted in a timely stable overexpression of the pH sensors. Transduced cells were seeded and cultivated according to the FLIM protocol. For imaging of NAD(P)H autofluorescence, a 460/60 and a 436/20 emission filters were both used to exclude Mito-SypHer to be detected in the FLIM channel. Mito-SypHer and Cyto-pHred were imaged first, followed by NAD(P)H FLIM both recorded as described above.

### 2.10 Biochemical NAD+/NADH and NADP+/NADPH Measurement

HEK293 cells were seeded in 24-well plates with glass bottom in a culture medium supplemented with 10% FCS, 1% P/S, and 25 mM HEPES (pH: 7.4) at a similar concentration as for NAD(P)H FLIM. One NAD(P)H FLIM image was taken to control for the effect of the treatments. Afterward, cells were immediately lysed in 1% DTAB in 0.2 M NaOH upon the recommendation of the NAD/NADH-GloTM Assay’s manual. For separation of $\mathrm{NAD}{\left(P\right)}^{+}$ and NAD(P)H, the lysate of one well was split and heated to 60°C for 15 min under alkaline or acidic condition also according to the NAD/NADH-GloTM Assay’s manual. Subsequently, the NAD/NADH-GloTM Assay (Promega) and the NADP/NADPH-GloTM Assay (Promega) were performed according to the manufacturer’s protocol.

### 2.11 Statistical Analysis

Statistical analysis was performed using GraphPad Prism 5 (GraphPad Software, Inc.). D’Agostino & Pearson omnibus normality test was used to check for a Gaussian distribution of the data (significance level α=0.05). For data in which all groups passed the normality test, an unpaired two-tailed t-test (for two groups) or a one-way ANOVA using Bonferronis pairwise multiple comparison (for more than two groups) was performed to check for significance. Nonnormal distributed data were analyzed with a Mann–Whitney-U-test (two groups) or Kruskal–Wallis test and Dunn’s multiple comparison test (for more than two groups). The slope of linear regressions was calculated using an F-test. Significance levels were defined as 0.05 (*), 0.01 (**), and 0.001 (***) and are indicated in the graphs.

## 3 Results

### 3.1 Modification of Respiration by Single Chemicals Each Result in a Good Correlation of NAD(P)H FLIM and Cellular Respiration

**Fig. 1:**
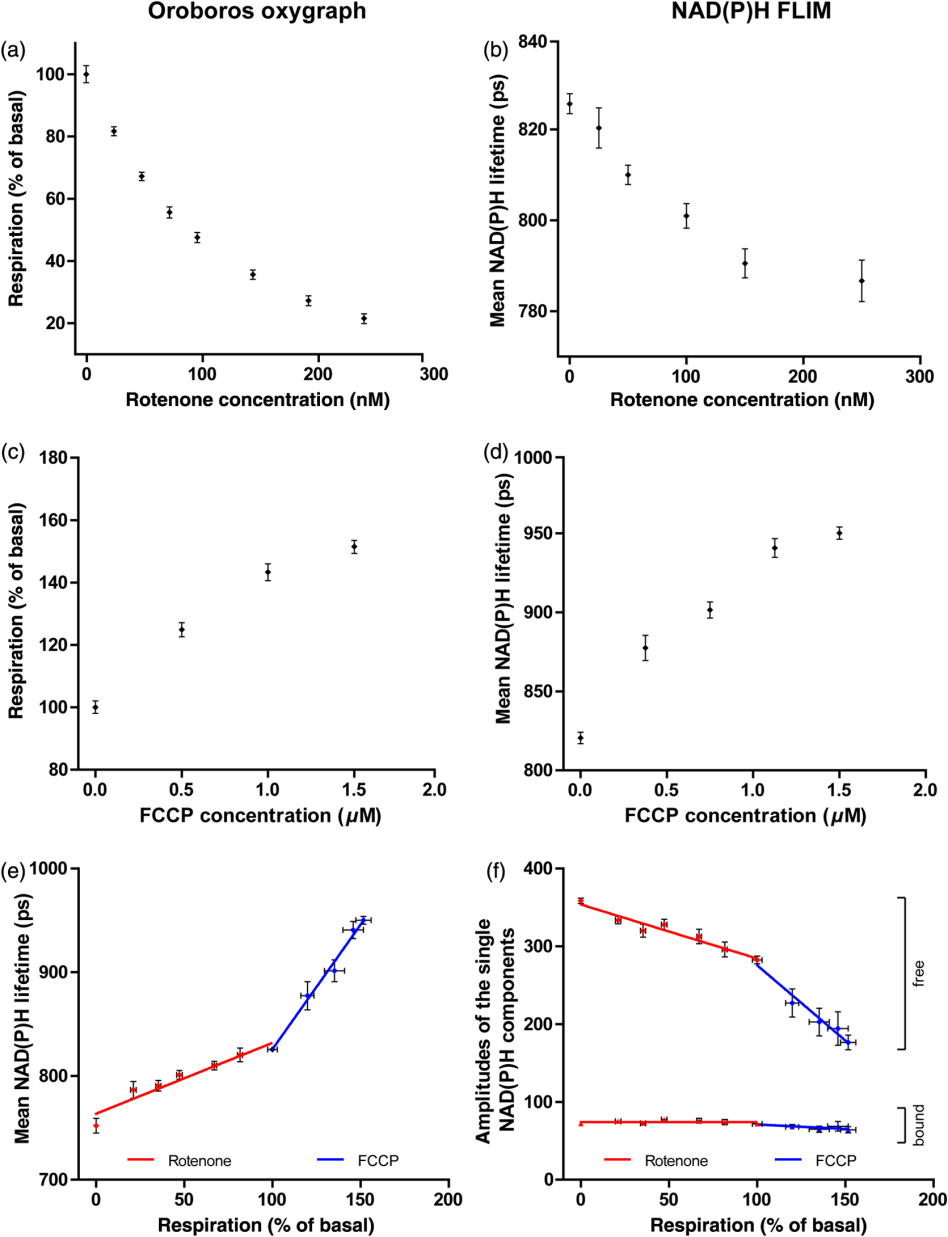
Quantitative correlation of respirometry and NAD(P)H FLIM. (a/c) High-resolution respirometry performed in intact HEK293 cells in an Oroboros Oxygraph-2k. Oxygen consumption of the cells was normalized to routine respiration, which was set to 100%. The means of seven independent experiments, each measured in duplicate, are shown. Error bars indicate standard error. (b/d) Quantification of the NAD(P)H fluorescence lifetime after titrations of (b) rotenone or (d) FCCP in HEK293 cells determined by NAD(P)H FLIM in a conditioned medium at 37°C with ambient ${\mathrm{CO}}_{2}$. At lower rotenone concentrations, cells were given 15-min adaption time after injection, according to the delayed effect observed in the Oroboros Oxygraph. Means of (b) three or (d) four independent experiments, each with 50 cells per condition, are shown. Error bars indicate standard error. (e) Combination of (a)–(d) reveals respiration in percent of routine respiration plotted against NAD(P)H lifetime. Blue data points indicate that respiration was modified by FCCP and red indicates a modification by rotenone. Linear regressions were calculated, displaying a significantly different slope (p≤0.0001). (f) The absolute amplitude of the single lifetime components (a1abs and a2abs) plotted over the percent of routine respiration. Diamonds display free NAD(P)H and triangles code for bound-NAD(P)H.

First, an unbiased, quantitative correlation of NAD(P)H lifetime, willingly not corrected for any influencing factors, with mitochondrial respiration was performed. This should reveal the goodness of “raw” NAD(P)H lifetime as a marker of mitochondrial respiration. We performed a titration of the complex I inhibitor rotenone to gradually inhibit NADH-based respiration in HEK293 cells. In our reference system, an Oroboros Oxygraph-2k, rotenone was titrated in, resulting in an exponential decay of respiration [Fig. 1(a)]. Likewise, the addition of rotenone to HEK293 cells measured by NAD(P)H FLIM resulted in an exponential decay of the mean NAD(P)H lifetime (τmean) [Fig. 1(b)]. To stimulate respiration, we added the uncoupler FCCP to HEK293 cells stepwise. By releasing the proton gradient, this results in a higher respiration, which was demonstrated using the Oroboros Oxygraph 2k [Fig. 1(c)]. Similarly, FCCP results in an increased NAD(P)H lifetime [Fig. 1(d)]. NAD(P)H fluorescence intensities also correlated with respiration; however in an antidromic manner (data not shown), indicating no marked differences of expressiveness of NAD(P)H lifetime and NAD(P)H intensity for assessing cell metabolism in monolayer cells. Plotting NAD(P)H lifetime over the percentage of routine respiration [Fig. 1(e)] reveals a linear dependence of NAD(P)H lifetime on cellular respiration for each, the rotenone and the FCCP titration (linear regression for rotenone: $r^{2}=0.9243$ and for FCCP: $r^{2}=0.9884$). However, the slope of both linear regressions differs significantly (p<0.0001). Assuming an exclusive dependence of the NAD(P)H lifetime on respiration, this dependence should be autonomous of how respiration is modified. Though, FCCP-induced respiratory modification has a stronger effect on NAD(P)H lifetime than rotenone-induced modification. This indicates a marked side effect of the chemicals on NAD(P)H lifetime independent of their metabolic effects. Analysis of the absolute amplitudes of the single NAD(P)H lifetime components revealed that mainly changes in the free NAD(P)H components account for the changes of the mean lifetime [Fig. 1(f)]. This indicates that the amount of protein-bound NAD(P)H is not influenced during respiratory alterations but the amount of free NAD(P)H is increasing or decreasing. However, the difference in the slope of both linear regression can be seen also in this diagram. To summarize, NAD(P)H lifetime is linearly dependent on respiration but seems to be further influenced by other cellular processes associated with these metabolic changes.

### 3.2 Uncoupler Inhibitor Combinations Reveal Limitations of NAD(P)H FLIM in Detecting Cell Metabolism

**Fig. 2:**
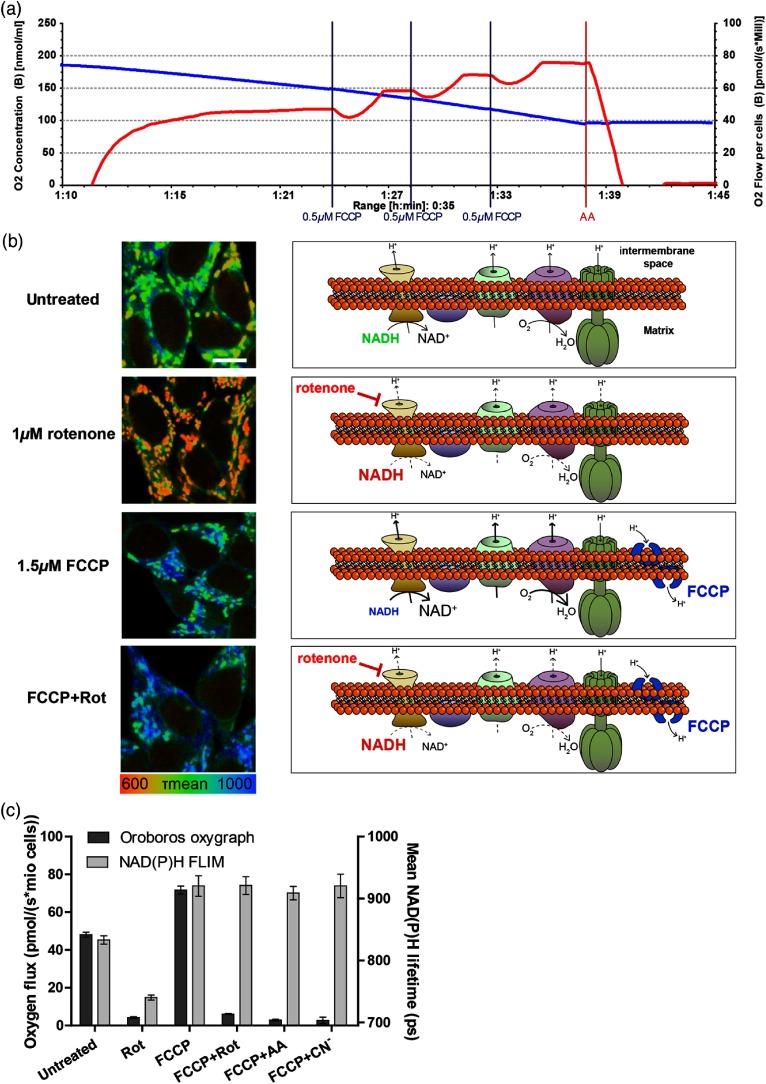
Uncoupler–inhibitor protocols reveal deviations in respirometry and NAD(P)H FLIM. (a) Representative uncoupler–inhibitor protocol for intact HEK293 cells in an Oroboros Oxygraph-2k with the blue line indicating the oxygen concentration and the red line the oxygen flux. Vertical lines indicate addition of a substance [three times 0.5-μM FCCP, and 5-μM antimycin A (AA)] (b) On the right-hand side, the effects of FCCP, rotenone, and the combination on the ETS activity is displayed. Derived from these, the expected consequences on NADH are depicted as the size (autofluorescence intensity) and color (lifetime/red = short and blue = long) of the word NADH. On the left, representative NAD(P)H FLIM image sections of HEK293 cells treated with those chemicals are displayed. The mean lifetime of NAD(P)H is coded in false-colors. Corresponding color palette is shown below. The white bar has a length of 10  μm. (c) Comparison of the oxygen flux (dark gray bars, left y-axis) and NAD(P)H FLIM (light gray bars, right y-axis) of HEK293 cells treated with an inhibitor [1-mM potassium cyanide (${\mathrm{CN}}^{-}$), 5-μM antimycin A, and 1-μM rotenone] or uncoupler (1.5-μM FCCP) of the mitochondrial respiratory system or a combination. At least three independent experiments were performed, each measured in duplicate for respirometry or for NAD(P)H FLIM each with 50 cells imaged per condition. Error bars indicate standard error.

**Fig. 3:**
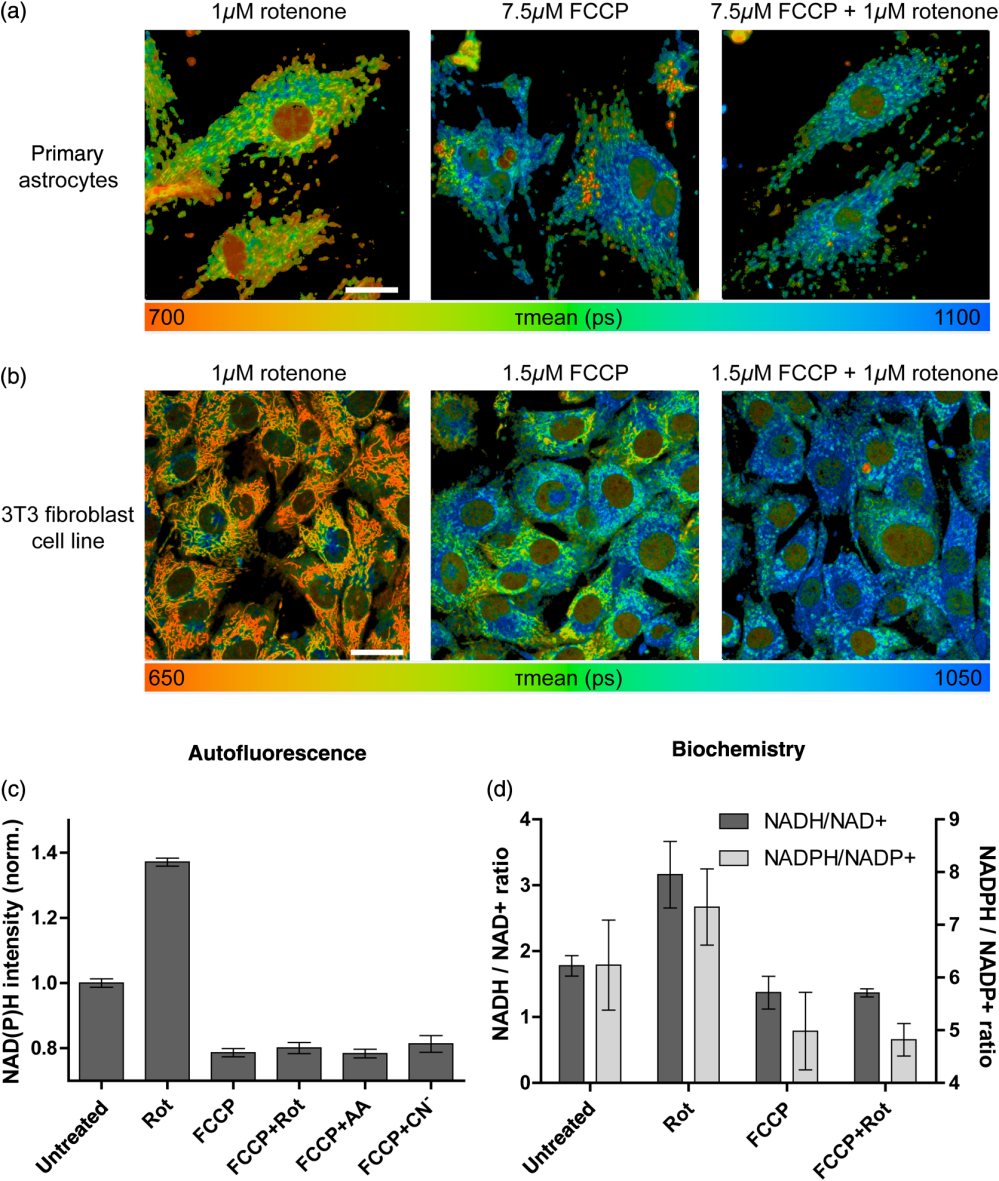
Uncoupler–inhibitor combinations in NAD(P)H FLIM of various cells. (a/b) Representative image sections of NAD(P)H FLIM of primary astrocytes (a) and 3T3 fibroblast cells (b) treated with FCCP, rotenone or the combination of both in their conditioned culture medium. The mean lifetime of NAD(P)H is false-color coded with the corresponding color-palette to be shown below the images. Lifetimes were calculated using a biexponential decay with lifetimes fixed to 400 and 2500 ps in SPCImage 5.0. The white scale bar has a length of 20  μm. (c) NAD(P)H intensity of HEK293 cells treated with an inhibitor [1-mM potassium cyanide (${\mathrm{CN}}^{-}$), 5-μM antimycin A, and 1-μM rotenone] or uncoupler (1.5-μM FCCP) of the mitochondrial respiratory system or a combination. At least three independent experiments were performed, each with 50 cells imaged per condition. Error bars indicate standard error. (d) Biochemically measured NADH (dark gray bars, left y-axis) and NADPH (light gray bars, right y-axis) redox state of HEK293 cells upon uncoupling and blockage of respiration. Three independent experiments were performed, each with three biological replicates. Error bars indicate standard error.

To further investigate alterations in NAD(P)H lifetime following uncoupling, we applied uncoupler–inhibitor protocols. A typical substance combination for metabolic characterization of intact cells in high-resolution respirometry is shown in Fig. 2(a), where the red curve displays respiration.^34^ Starting with routine respiration, first FCCP (1.5  μM) is added to reveal the electron transport system (ETS) capacity and subsequently respiration is blocked for example by the complex III inhibitor antimycin A (5  μM). The changes in ETS activity upon treatment with FCCP, the complex I inhibitor rotenone (1  μM) or a combination of both are displayed on the right-hand side in Fig. 2(b). In addition, the color of “NADH” indicates the expected changes in NAD(P)H lifetime with red indicating a shorter lifetime and blue indicating a longer lifetime. As soon as 1  μM of rotenone is present, the CI-mediated electron flow is stopped, independent on the presence of an uncoupler such as FCCP.^34^ Thus, the free NADH is not oxidized anymore and accumulates in the matrix, what should result in a shorter NAD(P)H lifetime.^5^ Representative NAD(P)H FLIM images of HEK293 cells treated with these compounds are displayed on the left-hand side [Fig. 2(b)]. As expected and already observed in the titrations in Fig. 1, rotenone reduces and FCCP increases NAD(P)H lifetime. Though, in contrast to the expectations, FCCP+rotenone results in a very long NAD(P)H lifetime. A quantification of the NAD(P)H lifetime [Fig. 2(c), light gray bars] and also of the respective oxygen flux [Fig. 2(c), dark gray bars] in HEK293 cells confirmed the observation. For the inhibitor–uncoupler combination, NAD(P)H FLIM does obviously not mirror cellular respiration [Fig. 2(c)]. This is not only true for complex I inhibition with rotenone (1  μM) but we tested several treatment combinations of FCCP with different inhibitors of the mitochondrial respiratory system (complex III by 5-μM antimycin A or complex IV by 1-mM cyanide). All display a clear deviation of mitochondrial respiration and NAD(P)H lifetime. Regarding the representative images [Fig. 2(b), left], it becomes evident that in all conditions, primarily the mitochondrial, NAD(P)H undergoes the alterations. This excludes the idea of other NAD(P)H pools causing this side effect. In addition, also applying FCCP after blockage of respiration results in a long NAD(P)H lifetime (data not shown). To exclude a cell line-specific side effect, we tested a range of different cells with the uncoupler–inhibitor combination. All showed lower NAD(P)H autofluorescence intensity and long NAD(P)H lifetimes following this treatment [Figs. 3(a) and 3(b)]. To further check whether this lifetime alteration does also manifest on the redox state, we measured the ${\mathrm{NAD}}^{+}/\mathrm{NADH}$ redox ratio biochemically, using a kit that is specific for ${\mathrm{NAD}}^{+}/\mathrm{NADH}$ versus ${\mathrm{NADP}}^{+}/\mathrm{NADPH}$. Similarly, the combination of FCCP and rotenone results in a shift toward ${\mathrm{NAD}}^{+}$ [Fig. 3(d), dark gray bars], whereas blockage of respiration without uncoupling entails a shift toward NADH. This demonstrates that the observed phenomenon is not only imaging-associated and that NADPH cannot be the responsible disturbing factor. Indeed, the ${\mathrm{NADP}}^{+}/\mathrm{NADPH}$ redox couple shifted toward ${\mathrm{NADP}}^{+}$ for the uncoupler–inhibitor combination, which cannot explain the unexpected long NAD(P)H lifetime.

To sum up, metabolic imaging using NAD(P)H does not work under certain conditions, especially when the mitochondria are in an uncoupled state. Besides increasing respiration, the second prominent effect of uncoupling is the reduction of the proton motive force [mitochondrial membrane potential (mtΨ) and pH-gradient] across the inner mitochondrial membrane. Thus, we hypothesized one of those to be the disturbing factor.

### 3.3 Alterations in Mitochondrial Matrix pH Distort the Metabolic Imaging by NAD(P)H FLIM

**Fig. 4:**
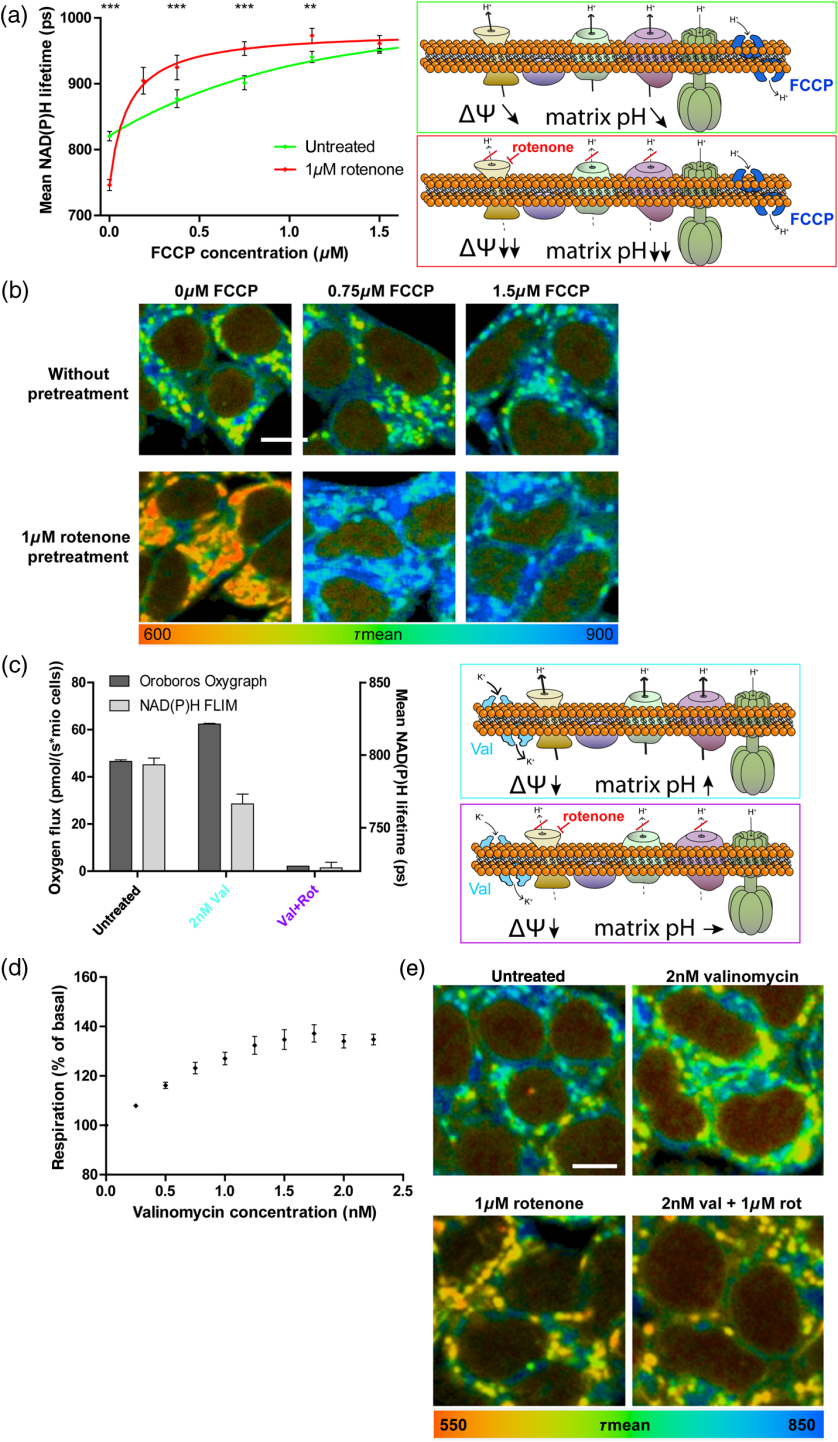
NAD(P)H lifetime is influenced by respiration and pH. (a) Quantitative analysis of NAD(P)H lifetime after an FCCP-titration in HEK293 cells pretreated with mock (green) or rotenone (red). Means of four independent experiments, each with 50 cells per condition, are displayed. Error bars indicate standard error. Significance versus control was evaluated using the Mann–Whitney test (*p<0.05). The effects of an increasing FCCP concentration on the proton motive force are drafted on the right-hand side. (b) Representative NAD(P)H FLIM image sections of HEK293 cells pretreated with mock or 1  μM rotenone after the addition of 0, 0.75, or 1.5  μM of FCCP. NAD(P)H lifetime is coded in false-colors as indicated below. The white bar has a length of 10  μm. (c) Comparison of the oxygen flux (dark gray bars, left y-axis) and NAD(P)H FLIM (light gray bars, right y-axis) of HEK293 cells treated with valinomycin±rotenone. Two independent experiments were performed in respirometry, each measured in duplicate and three independent experiments are shown for NADH FLIM, each with 50 cells analyzed per condition. Error bars indicate standard error. On the right-hand side, the mode of action and the consequences on the proton motive force are drafted. (d) High-resolution respirometry performed in intact HEK293 cells in an Oroboros Oxygraph-2k. Titration protocol: addition of 2mio cells in their conditioned medium (routine respiration), titration of valinomycin to a final concentration of 2.25 nM, addition of 1  μM rotenone, and 5  μM antimycin A (ROX). Oxygen consumption of the cells was corrected for ROX and normalized to the routine respiration, which was set to 100%. The means of two independent experiments, each measured in duplicate, are shown. Error bars indicate standard error. (e) Representative image sections of NADH FLIM of HEK293 cells treated with valinomycin, rotenone, or the combination of both in their conditioned culture medium. The color code displays the mean lifetime of NADH. The corresponding color-palette is shown below the images. The white scale bar has a length of 10  μm.

We hypothesized that the dissipation of the proton motive force itself caused the aberrant long NAD(P)H lifetime following the uncoupler–inhibitor combination. Indeed, when the pumping of protons to the intermembrane space is blocked, for example by rotenone pretreatment, increasing concentrations of FCCP results in an earlier/faster dissipation of the proton motive force [Fig. 4(a), right]. Accordingly, one would expect an earlier/faster elongation of NAD(P)H lifetime in rotenone pretreated cells upon an FCCP titration.

Thus, we performed this titration of FCCP in HEK293 cells after pretreatment with rotenone to check for our hypothesis of mtΨ or matrix pH to be the disturbing factor for metabolic NAD(P)H FLIM. Indeed, pretreatment with rotenone results in a significantly longer NAD(P)H lifetime at low FCCP concentrations [Fig. 4(a), left]. This marked difference can also be demonstrated visually regarding the microscopic images [Fig. 4(b)]. This result strongly indicates that the disturbing factor is associated to the proton motive force. To further underline this finding and to differentiate the influence of mitochondrial matrix pH and mitochondrial membrane potential on NAD(P)H lifetime, we applied valinomycin on HEK293 cells. Valinomycin is an ionophore with a high specificity for potassium ions, thereby dissipating the electrical gradient over the inner mitochondrial membrane but increasing the chemical proton gradient [Fig. 4(c), right].^35^ Thus, similar to FCCP, it stimulates respiration in HEK293 cells [Fig. 4(c)] and reduces the mitochondrial membrane potential. Comparing the effect of 2 nM of valinomycin on respiration as well as on NAD(P)H lifetime, we observed increased respiration but shorter NAD(P)H lifetime [Fig. 4(c)], mainly in the mitochondria-rich region [Figs. 4(d) and 4(e)]. Thus, valinomycin shows a contrary effect on NAD(P)H lifetime compared to FCCP, indicating matrix pH to be a determinant of NAD(P)H lifetime. This further goes along with the hypothesis of a more alkaline mitochondrial pH shortening NAD(P)H lifetime in the mitochondria, whereas acidification (after FCCP and FCCP+Rot) caused a long NAD(P)H lifetime.

### 3.4 Quantification of NAD(P)H Lifetime Dependence on Intracellular pH

**Fig. 5:**
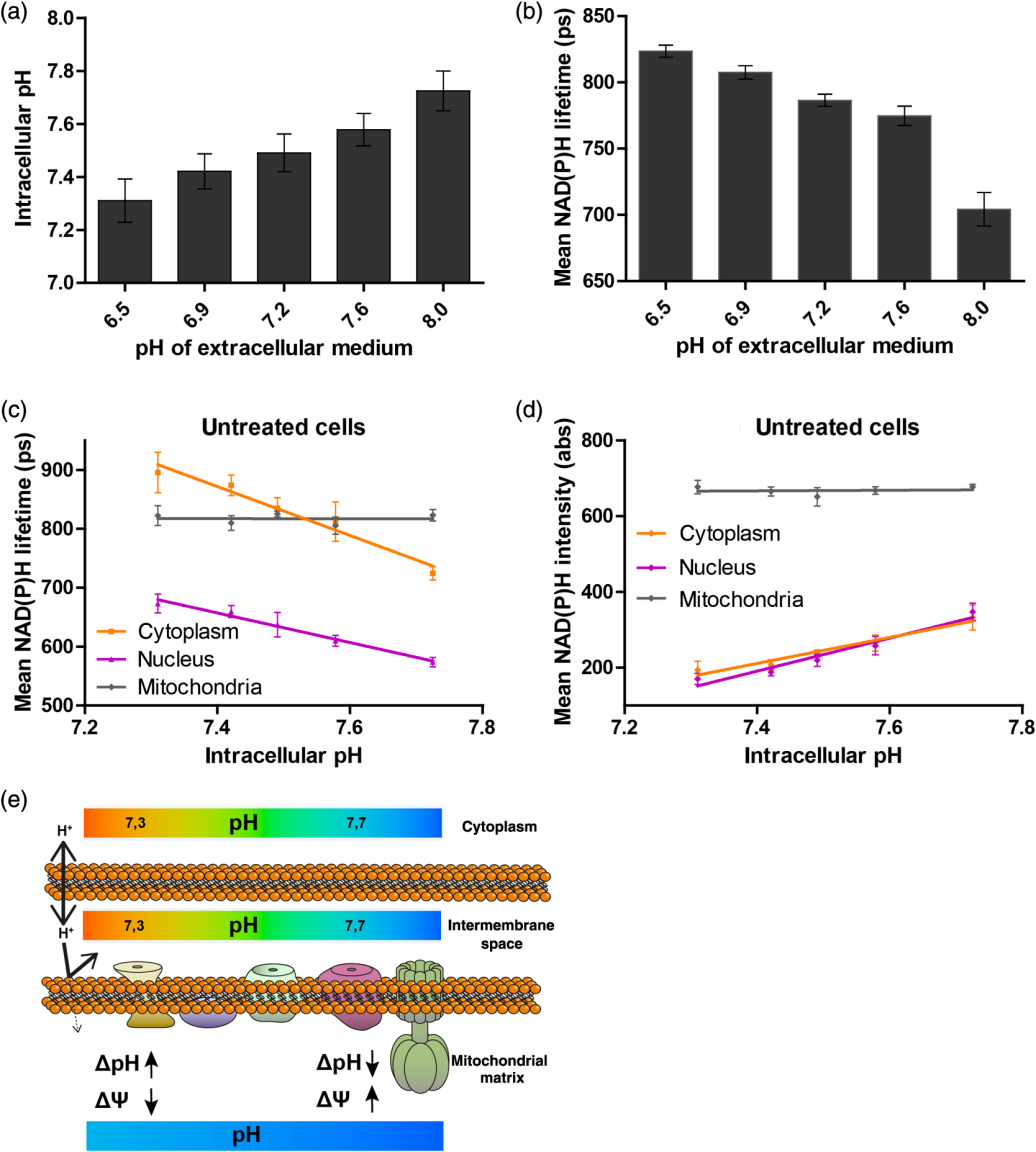
Quantitative assessment of the influence of pH on NAD(P)H lifetime in cellular compartments. (a) Mean intracellular pH of untreated, intact HEK293 cells in a medium with different pH values determined microscopically using BCECF-AM. Four independent experiments were performed, each with 50 cells analyzed per condition. Error bars indicate standard error. (b) NAD(P)H lifetime of untreated, intact HEK293 cells in a medium with different pH values. At least three independent experiments were performed, each with 50 cells analyzed per condition. Error bars indicate standard error. (c) Subcellular analysis of nuclear (purple), cytoplasmic (orange), or mitochondrial (gray) NAD(P)H lifetime alterations with intracellular pH. A representative experiment of (b) was analyzed with at least 50 cells per condition. Error bars indicate standard error. Linear regressions were calculated, shown by the colored lines. (d) Subcellular analysis of nuclear (purple), cytoplasmic (orange), or mitochondrial (gray) NAD(P)H autofluorescence intensity alterations with intracellular pH. A representative experiment of (b) was analyzed with at least 50 cells per condition. Error bars indicate standard error. Linear regressions were calculated, shown by the colored lines. (e) Draft of the impact of changing intracellular pH on the mitochondrial proton motive force and matrix pH. Whereas the outer mitochondrial membrane is permeable for protons, the IMM is not. Accordingly, the mitochondrial matrix pH remains rather stable and there is mainly a shift in the components of the proton motive force.

To quantify the impact of alterations in intracellular pH on the NAD(P)H lifetime, we performed NAD(P)H FLIM of untreated HEK293 cells, varying extracellular medium pH. Increasing extracellular pH resulted in an alleviated increase in intracellular pH [Fig. 5(a)] as determined by a pH-sensitive dye named BCECF-AM. Likewise, we observed a decrease in the mean NAD(P)H lifetime [Fig. 5(b)], which goes in line with our previous observations of a more alkaline pH shortening NAD(P)H lifetime. As this effect size might be influenced by the protein composition in the cellular compartments, we performed a subcellular analysis on the NAD(P)H lifetime and intensity level. This revealed that nuclear as well as cytosolic NAD(P)H change along with the intracellular pH [Figs. 5(c) and 5(d)]. In contrast, mitochondrial NAD(P)H is not affected. A possible reason could be that the inner mitochondrial membrane is impermeable for proton diffusion, shielding the mitochondrial matrix pH against intracellular pH changes in untreated cells. Accordingly, a more acidic intracellular pH would result in a higher ΔpH in exchange for a lower ΔΨ [Fig. 5(e)].

### 3.5 Determining the Effect Size of pH on NAD(P)H Lifetime in Mitochondria

**Fig. 6:**
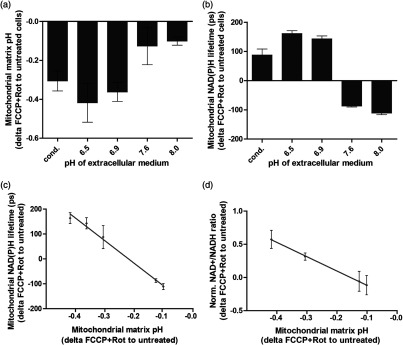
Quantitative assessment of the influence of pH on NADH in mitochondria. (a) Alterations of mitochondrial matrix pH following the uncoupler–inhibitor combination FCCP [(1.5  μM) + rotenone (1  μM)] in HEK293 cells overexpressing mito-SypHer cells. Cells were measured microscopically in a medium with different pH, which was kept stable using 25 mM HEPES. “Cond.” Indicates a conditioned medium with an original pH of 7.4. The means of four independent experiments, each with 50 cells per condition, are shown. Error bars indicate standard error. (b) Alterations of mitochondrial NAD(P)H lifetime following the uncoupler–inhibitor combination FCCP [(1.5  μM) + rotenone (1  μM)] in HEK293 cells. The means of three independent experiments, each with 50 cells per condition, are shown. Error bars indicate standard error. (c) Changes of mitochondrial NAD(P)H lifetime (b) correlated with changes of mitochondrial matrix pH (a) upon treatment with 1.5-μM FCCP plus 1-μM rotenone in HEK 293 cells in medium with different pH values. A linear regression was calculated. (d) Changes of NADH redox state with mitochondrial matrix pH upon treatment with 1.5-μM FCCP plus 1-μM rotenone in HEK 293 cells in medium with different pH values. Three independent experiments for NADH redox state were performed, each with two biological duplicates. Error bars indicate standard error. A linear regression was calculated.

To determine the respective mitochondrial matrix pH, we overexpressed the pH-sensitive mito-SypHer in HEK293 cells. We could demonstrate that administration of FCCP+Rot, which allows us to overcome the proton barrier function of the inner mitochondrial membrane, results in a pronounced reduction of the mitochondrial matrix pH when cells were incubated at an acidic extracellular pH. In contrast to this, the acidification of the mitochondrial matrix was rather low in a more alkaline environment upon FCCP+Rot treatment [Fig. 6(a)]. This confirms our assumptions made in Fig. 5(e) and further allows us to evaluate the changes of mitochondrial NAD(P)H with mitochondrial matrix pH. To do so, we measured the alteration of mitochondrial NAD(P)H lifetime upon treatment with FCCP+Rot. At more acidic pH values, we observed an elongation of NAD(P)H lifetime [Fig. 6(b)], as already described before [see Fig. 2(c)]. In contrast, at a more alkaline pH, NAD(P)H lifetime shortens as it would be expected for a treatment that blocks respiration [Fig. 6(b)]. To summarize, there is a clear trend that NAD(P)H lifetime resembles respiration better when there are only minor changes in matrix pH.

Plotting the mitochondrial NAD(P)H lifetime alteration [Fig. 6(b), y-axis] over the respective mitochondrial matrix pH changes [Fig. 6(a), y-axis] reveals the dependence of NAD(P)H lifetime on the pH in mitochondria [Fig. 6(c)]. Interestingly, calculating a linear regression, it exhibits twice the slope as compared to the linear regression calculated for cytoplasm or nucleus [see Fig. 5(c)]. Accordingly, changes in mitochondrial matrix pH have a stronger impact on NAD(P)H lifetime than cytosolic or nuclear pH changes. Whether this is due to the protein composition remains pending, but in combination with matrix pH being the most variable,^36^ this underlines the importance of matrix pH as an influencing factor in metabolic imaging. To ensure that it is really the NADH that shows the pH dependence and not another factor such as NADPH, we also checked for the NADH redox state biochemically. Similarly, a clear pH dependence of the ${\mathrm{NAD}}^{+}/\mathrm{NADH}$ redox state is evident [Fig. 6(d)]. This demonstrates that mitochondrial NADH is strongly altered by the mitochondrial matrix pH, which consequently is a key factor to be considered for quantification of mitochondrial respiration by NAD(P)H FLIM.

### 3.6 Correction for Mitochondrial pH Results in a Linear Correlation of NAD(P)H Lifetime and Respiration

**Fig. 7:**
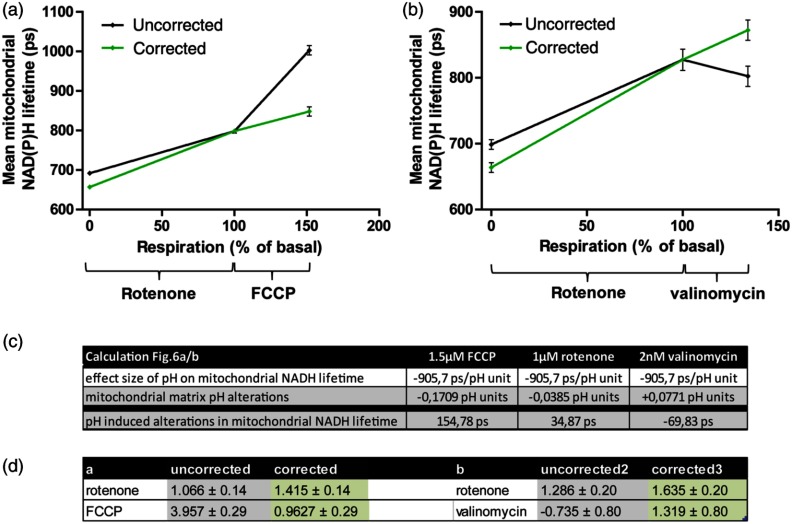
Correction for mitochondrial matrix pH reveals a linear dependence of mitochondrial NAD(P)H lifetime on cellular respiration. (a) The end points of the FCCP and rotenone titrations in NAD(P)H FLIM [see Figs. 1(b) and 1(d)] were analyzed subcellularly for mitochondrial NAD(P)H lifetimes and compared to respiration [see Figs. 1(a) and 1(c)], resulting in the black rhombs. The green rhombs represent the NAD(P)H lifetime values corrected for the mitochondrial matrix pH-induced lifetime alterations in the respective conditions. Separate linear regressions for the FCCP and the rotenone part were added according to the observations in Fig. 1. Means of at least three independent experiments, each with 50 cells per condition, are shown. Error bars indicate standard error. (b) NAD(P)H FLIM experiments of valinomycin and valinomycin plus rotenone [see Fig. 4(c)] were analyzed subcellularly for mitochondrial NAD(P)H lifetimes and compared to respiration, resulting in the black rhombs. The green rhombs represent the NAD(P)H lifetime values corrected for the mitochondrial matrix pH-induced lifetime alterations in the respective conditions. Means of at least three independent experiments, each with 50 cells per condition, are shown. Error bars indicate standard error. (c) Calculation of the pH-induced lifetime alteration as matrix pH alteration (n=4) multiplied by the effect size of pH on NAD(P)H lifetime [slope of the linear regression in Fig. 6(c)]. (d) Correction for pH-induced alterations of NAD(P)H lifetime (slopes of Fig. 7).

In order to validate the quantitative impact of pH alterations on NAD(P)H FLIM, we corrected NAD(P)H lifetime for the effect of mitochondrial matrix pH alterations in the correlations of respiration and NAD(P)H FLIM [see Fig. 1(e)]. For this purpose, we determined the alterations in mitochondrial matrix pH following treatment with 1.5  μM FCCP, 1  μM rotenone or 2 nM valinomycin in HEK293 cells overexpressing mito-SypHer [Fig. 7(d)]. In addition, respective NAD(P)H FLIM measurements were subcellularly analyzed for mitochondrial-rich regions [Figs. 7(a) and 7(b)]. As for each single substance, the titrations showed linear characteristics [see Fig. 1(e)], the subcellularly analyzed endpoints were connected by a line. To correct these lifetimes for the impact of pH, alterations in mitochondrial matrix pH were multiplied by the effect size of pH in mitochondria [Fig. 7(c)]. The black lines in Figs. 7(a) and 7(b) display the uncorrected correlations of mitochondrial NAD(P)H lifetime and respiration. After correction for pH-induced alterations, the green lines reveal an almost linear dependence of NAD(P)H lifetime and respiration, regardless by which chemicals the respiration is modified [Fig. 7(d)]. This demonstrates that correction for mitochondrial matrix pH advances NAD(P)H FLIM as quantitative marker for mitochondrial respiration.

### 3.7 Imaging Mitochondrial Function in a Pathological Context of Alzheimer’s Disease by NAD(P)H FLIM

**Fig. 8:**
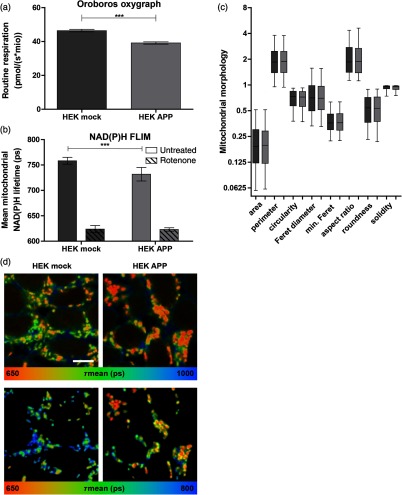
Mitochondrial function in an APP-overexpressing model of Alzheimer’s disease. (a) High-resolution respirometry performed in intact HEK293 cells overexpressing APP or a control vector in an Oroboros Oxygraph-2k. The means of eight independent experiments, each measured in duplicate, are shown. Error bars indicate standard error. Significance was evaluated using Mann–Whitney-U-test. (b) pH-corrected NAD(P)H fluorescence lifetime in stably transduced HEK293 cells. Mean mitochondrial NAD(P)H lifetime of six independent experiments with at least 50 cells per experiment. Respiration was blocked by 1-μM rotenone. For mitochondrial matrix pH correction, six independent experiments were performed using FACS analysis and NAD(P)H lifetime was corrected for pH-induced lifetime alterations [calculated as described in Fig. 7(c)]. Error bars indicate standard error. Significance was evaluated using t-test with Welch correction for unequal variance. (c) Mitochondrial morphology analyzed by TEM. Sizes are displayed in the μm-range. Whiskers indicate 5 to 95 percentile. Three independent experiments are shown with more than 500 mitochondria analyzed per condition. (d) Representative image sections of (b) displaying the mean lifetime of NAD(P)H coded in false-colors. Corresponding color palette is shown below. The white bars have a length of 10  μm. The upper panel shows the whole cells and the lower panel is analyzed for mitochondrial NAD(P)H.

To prove the suitability and sensitivity of NAD(P)H FLIM in analyzing mitochondrial function in a pathological context, we applied it on an APP-overexpressing model of Alzheimer’s disease (AD). HEK293 cells stably transduced with a control plasmid (pUltra-hot) or an APP-expressing plasmid (APP pUltra-hot) were first analyzed for their routine respiration in an Oroboros Oxygraph-2k. HEK APP displayed a reduction in routine respiration of about 15% compared to control cells [Fig. 8(a)]. In NAD(P)H FLIM, already corrected for pH-induced lifetime shift, untreated HEK APP cells showed a significantly shorter mitochondrial NAD(P)H lifetime [Fig. 8(b)]. To further underline this lifetime difference to originate from alterations in respiration, NAD(P)H lifetimes for rotenone-treated cells are displayed [Fig. 8(b), dashed bars], which do not differ significantly. This emphasizes the sensitivity of NAD(P)H FLIM to detect even slight mitochondrial defects in a pathophysiological model. According to a potential morphofunctional association, we analyzed whether mitochondrial morphology is altered by APP-overexpression using transmission electron microscopy. However, we did not detect any morphological differences between APP-overexpressing and control HEK293 cells [Fig. 8(c)], indicating that a functional deficit does not necessarily influence mitochondrial morphology. In contrast, representative NAD(P)H lifetime images of untreated HEK APP and control cells [Fig. 8(d)] illustrate the altered mitochondrial function on the subcellular level. This highlights NAD(P)H FLIM complemented with matrix pH measurements as a unique microscopic tool to investigate mitochondrial health and to detect subtle changes in respiratory activity on the mitochondrial level.

### 3.8 Parallel Measurement of NAD(P)H FLIM and pH Reveal Energy Metabolism on the Single Cell Level

**Fig. 9:**
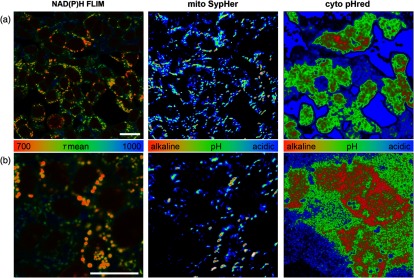
Parallel measure of pH and NAD(P)H FLIM for metabolic imaging. Representative image sections of parallel measurements of NAD(P)H lifetime, cytosolic pH using pHred and mitochondrial matrix pH using SypHer in HEK293 cells treated with 1.5-μM FCCP and 1-μM rotenone in medium with pH 7.2. (a) An overview image at lower magnification to allow a correlation of intracellular pH to NAD(P)H lifetime. (b) The image section with a higher magnification demonstrates the pH-NAD(P)H lifetime correlation even on the mitochondrial level. NAD(P)H lifetime is coded in false-colors with red indicating shorter lifetimes. Ratios of the emissions at the different excitation wavelength of the pH-sensitive dyes are false-color coded with red indicating a more alkaline pH. The white bars have a length of 20  μm.

To make full use of the high spatial resolution of NAD(P)H FLIM, it is essential to measure both, NAD(P)H lifetime and pH, in the very same cells. We created an expression construct for a combination of two pH-sensitive dyes, with SypHer targeted to the mitochondrial matrix and pHred remaining in the cytosol. HEK293 cells were transduced, and cells were imaged first by snap exposures at 405, 488, and 561 nm for the pH indicators followed by a 1-min FLIM acquisition. NAD(P)H FLIM was not affected by the overexpression of the dyes or the previous snap exposures (data not shown). To demonstrate the capability of these parallel measurements, we imaged HEK293 cells in the medium with pH 7.2 and treated with 1.5-μM FCCP and 1-μM rotenone. Thus, the intracellular pH determines the reaction of NAD(P)H to this uncoupler–inhibitor combination. Under these conditions, both short and long NAD(P)H lifetimes can occur, varying from cell to cell. In Fig. 9, a single-cell correlation of NAD(P)H lifetime and cytosolic/nuclear as well as mitochondrial pH can be observed visually. This highlights the spatial resolution that can be achieved and underlines the potential of metabolic imaging assessing NAD(P)H lifetime and pH in parallel.

## 4 Discussion

NAD(P)H redox state is known and was used as a metabolic indicator for decades,^37^ being advanced by the use of NAD(P)H fluorescence lifetime as a more precise readout.^8^ Technical progress in TCSPC further raised accuracy,^38^ rendering it possible to promote a quantitative assessment of cellular energy metabolism by NAD(P)H FLIM. However, this brings into play confounding factors, as it is known that NAD(P)H lifetime is sensitive to a range of factors such as protein composition,^39^ NADPH levels,^40^^,^^41^ pH,^42^ and other ion concentrations.^43^ Here we demonstrate an unbiased correlation of uncorrected NAD(P)H lifetime and respirometry in an Oroboros Oxygraph in HEK293 cells. This cell model was chosen as HEK293 cells can be cultured adherently and in suspension, making metabolic measurements in the Oxygraph (cells in suspension) and in the microscope (cells adherent) the most comparable. We decided to use the Oroboros Oxygraph instead of an XF Analyzer from Seahorse Bioscience due to a higher flexibility with the number of injections and a higher precision for small changes in respiration.^44^ By chemically modifying respiration, we showed that NAD(P)H lifetime does not purely correlate with respiration, especially when using the uncoupler FCCP. Regarding the possible confounding factors in this situation, protein composition is not altered on the minute scale our experimental treatments were performed. NADPH is unlikely to cause the aberrant long NAD(P)H lifetime, as uncoupling dissipates membrane potential, resulting in a conversion of $\mathrm{NADPH}+{\mathrm{NAD}}^{+}$ to $\mathrm{NADH}+{\mathrm{NADP}}^{+}$. Accordingly, we showed NADPH levels to decrease under this condition. Furthermore, we observed the uncoupling effect also on the biochemically measured NADH redox ratio, excluding a purely imaging-associated effect.

We demonstrate that the proton gradient, released by uncoupling, and subsequent acidification accounts for the longer NAD(P)H lifetime. As our FCCP concentrations are within a range that still stimulates respiration, the respiratory complexes still pump protons, resulting in only a small mitochondrial acidification. Blockage of the proton pumping by a respiratory inhibitor enhances acidification of the mitochondrial matrix [compare Fig. 6(a)], exaggerating the deviation of respiration and NAD(P)H lifetime. This goes in line with the idea of mitochondrial matrix pH being a major confounding factor when using NAD(P)H autofluorescence as a marker of respiration. Our finding is underlined by Ogikubo et al.^42^ who used NAD(P)H lifetime for intracellular pH sensing and Drozdowicz-Tomsia et al. who mentioned an effect of uncoupling on NAD(P)H lifetime.^45^ To further understand the observed effect of the pH, it is worthwhile having a closer look on the interconnection of NAD(P)H autofluorescence lifetime, intensity, and redox state. Although being associated to one another, they are not equal. NAD(P)H autofluorescence intensity is almost linear to the NAD(P)H concentration.^46^ This implies a direct correlation to the redox state as long as the NAD(P)+/NAD(P)H pool size is stable but not when for example ${\mathrm{NAD}}^{+}$ is consumed by sirtuins or PARPs.^47^ Mean NAD(P)H lifetime displays the ratio of free to protein-bound NAD(P)H. A stronger reduction of NAD(P)H results in a saturation of protein binding and thus mainly an elevation of the free NAD(P)H, though alterations in protein composition or a direct alteration of protein-binding by pH could also alter NAD(P)H lifetime independently of the redox state. However, we showed the pH effect not only on the lifetime but also on the intensity and biochemical level, excluding any bias by the readout method. For the quantification, we focused on the lifetime readout, as this is the most sustainable for future applications in tissue or even *in vivo*.

Our results strongly suggest that the associated implications for pH affecting NAD(P)H redox state and autofluorescence were underestimated in the past.

For metabolic regulation, this insight could provide the missing link in the compartment-specific regulation of ${\mathrm{NAD}}^{+}$. It was reported that matrix acidification stimulates Sirt3 activity.^48^ We now demonstrate a potential link, as acidification results in an increase in ${\mathrm{NAD}}^{+}$, which in turn activates the sirtuins. This is of major importance as ${\mathrm{NAD}}^{+}$ is reckoned as one of the most promising factors increasing lifespan and mitochondrial function.^49^ Understanding its regulation, for example by matrix pH as demonstrated in our paper, might give a start to therapeutic approaches.

For metabolic imaging, we clearly point out the necessity to consider pH changes. Mitochondrial matrix pH does not only vary when using chemicals to modify respiratory activity but also under physiological conditions. As the proton gradient is directly built up and released by the respiratory complexes, both respiration and mitochondrial matrix pH are inseparably connected. Consequently, our results help in understanding recent applications of NAD(P)H redox state, for example, in neuron-astrocyte cocultures where changes in NAD(P)H redox state occurred after modulation of uncoupling proteins.^50^ To allow for quantitative metabolic imaging, pH alterations not only have to be considered, but their effect has to be quantified. To our knowledge, we are the first to have systematically analyzed the impact of pH differences on NAD(P)H fluorescence lifetime in cellular compartments. However, there is a possibility that pH alterations entailed changes in cellular metabolism as described before.^51^^,^^52^ Although we cannot exclude this for glycolysis levels, we checked mitochondrial respiration to exclude major changes that could account for NAD(P)H lifetime alterations. In addition, the impact of pH alterations on mitochondrial NAD(P)H was performed in cells with blocked respiration, totally rendering impossible any respiratory differences to explain this effect. Interestingly, there were significant differences in the impact of pH in the cellular compartments, maybe due to variations in the protein content. In general, the effect of pH on fluorescence lifetimes seems to be dependent on a cellular environment.^53^ We found the strongest impact on mitochondrial NAD(P)H, further emphasizing the importance to correct for mitochondrial matrix pH. Accordingly, we demonstrate an almost linear dependence of respiration and mitochondrial NAD(P)H lifetime after correction for mitochondrial matrix pH (Fig. 7).

As mentioned above, NAD(P)H FLIM can be influenced by further parameters such as temperature, NADPH levels, or protein composition. In our model system, these influencing factors played a minor role and did not confound the correlation of NAD(P)H lifetime and respiration strongly. Temperature is easy to keep constant in mammalian systems and variations will influence energy metabolism of the cells much stronger than it will confound the detection system. NADPH has to be taken into account in systems that strongly differ in their NADH/NADPH balance.^54^ This occurs especially when comparing different cells metabolically using NAD(P)H FLIM and goes along with another important influencing factor, namely variations in protein composition. Consequently, for comparing different cell types to each other, a combination of the NAD(P)H redox index^12^ and an additional detection of changes in matrix pH might be a promising approach. This approach is based on the idea of taking into account the effect size of alterations of NAD(P)H upon metabolic modification. Similarly, we could show a significantly altered NAD(P)H lifetime of untreated HEK APP versus HEK mock but no difference in rotenone-treated cells. This indicates a metabolic origin of the NAD(P)H lifetime difference in contrast to a general NAD(P)H lifetime shift due to alterations in protein composition.

In our system, we modified respiration using uncouplers and inhibitors of the mitochondrial respiratory system. As to the physiological relevance, one should first consider which subcellular NAD(P)H pool is analyzed. For mitochondrial NAD(P)H, the readout is mainly a balance of NADH production in the citric acid cycle and NADH consumption of the respiratory system. For an impairment of the mitochondrial respiratory system the situation is clear, as the NADH consumption is stopped, but the low ATP/ADP ratio drives the citric acid cycle until a product inhibition, among others by NADH, occurs. In case of an altered ATP demand, the variation in the ATP-to-ADP ratio influence activity of the respiratory system as well as of the citric acid cycle in the same direction. Thus, the question arises whether such differences in respiration could be detected by our system. Indeed, uncoupling simulates high ADP/ATP for both, the respiratory system and the citric acid cycle. Though, this did not compromise the quantification of respiration by NAD(P)H FLIM after correction for pH. This puts forward that NAD(P)H FLIM should also be able to detect differences in mitochondrial respiration due to energy demand. However, with regard to a primary impairment of the citric acid cycle, resulting in a subsequent reduction of respiration, we assume NAD(P)H FLIM to be limited. Under this condition, NADH production is reduced whereas the oxidation by complex I is intact, resulting most likely in a longer NAD(P)H lifetime and NADH oxidation despite reduced respiration. Consequently, this scenario requires further investigation. We can conclude that NAD(P)H FLIM represents a quantitative tool, deemed to detect defects in the mitochondrial respiratory system on the subcellular level.

The sensitivity of our approach could be demonstrated in APP-overexpressing HEK293 cells, exhibiting a slight mitochondrial impairment.^55^ We were able to verify the respirometry results quantitatively by NAD(P)H FLIM. To date, several studies have estimated the heterogeneous mitochondrial function in a cell by morphological analysis^56^^,^^57^ as an association of mitochondrial morphology and function has been proposed.^58^^,^^59^ However, here we could not detect any morphological differences despite an altered respiration, indicating that this morphofunctional association does not prove true in every case or at least is less sensitive for small respiratory changes. Thus, in contrast to other microscopically determinable parameters such as morphology, NAD(P)H lifetime much better mirrors the functionality of mitochondria.

To sum up, our innovative combination of parallel NAD(P)H FLIM and mitochondrial matrix pH measurement is able to provide a functional mitochondrial monitoring with high spatial resolution. This will allow for example to dissect the mitochondrial heterogeneity within cells,^60^ such as synaptic mitochondria in AD. In addition to a precise respiratory analysis on the cellular level, also the coupling state and redox balance^61^ can be estimated, yielding a multiparametric conclusion of mitochondrial health.
